# Activation of Calcium-Sensing Receptor increases intracellular calcium and decreases cAMP and mTOR in PKD1 deficient cells

**DOI:** 10.1038/s41598-018-23732-5

**Published:** 2018-04-09

**Authors:** Annarita Di Mise, Grazia Tamma, Marianna Ranieri, Mariangela Centrone, Lambertus van den Heuvel, Djalila Mekahli, Elena N. Levtchenko, Giovanna Valenti

**Affiliations:** 1 Department of Biosciences, Biotechnologies and Biopharmaceutics, University of Bari, Bari, 70125 Italy; 2 Department of Pediatric Nephrology, Radboud University Nijmegen Medical Centre, Nijmegen, 6525 HP The Netherlands; 3 Department of Pediatric Nephrology, University Hospital Gasthuisberg, Leuven, 3000 Belgium; 4 Department of Development & Regeneration, University of Leuven (KU Leuven), Leuven, 3000 Belgium; 5 Istituto Nazionale di Biostrutture e Biosistemi, Roma, 00136 Italy; 6 Center of Excellence in Comparative Genomics (CEGBA), University of Bari, Bari, 70125 Italy

## Abstract

Clinical and fundamental research suggest that altered calcium and cAMP signaling might be the most proximal events in ADPKD pathogenesis. Cells from ADPKD cysts have a reduced resting cytosolic calcium [Ca^2+^]_i_ and increased cAMP levels. CaSR plays an essential role in regulating calcium homeostasis. Its activation is associated with [Ca^2+^]_i_ increase and cAMP decrease, making CaSR a possible therapeutic target. Human conditionally immortalized Proximal Tubular Epithelial cells (ciPTEC) with stable knockdown of PKD1 (ciPTEC-PC1KD) and ciPTEC generated from an ADPKD1 patient (ciPTEC-PC1Pt) were used as experimental tools. CaSR functional expression was confirmed by studies showing that the calcimimetic NPS-R568 induced a significant increase in [Ca^2+^]_i_ in ciPTEC-PC1KD and ciPTEC-PC1Pt. Resting [Ca^2+^]_i_ were significantly lower in ciPTEC-PC1KD with respect to ciPTECwt, confirming calcium dysregulation. As in native cyst cells, significantly higher cAMP levels and mTOR activity were found in ciPTEC-PC1KD compared to ciPTECwt. Of note, NPS-R568 treatment significantly reduced intracellular cAMP and mTOR activity in ciPTEC-PC1KD and ciPTEC-PC1Pt. To conclude, we demonstrated that selective CaSR activation in human ciPTEC carrying PKD1 mutation increases [Ca^2+^]_i_, reduces intracellular cAMP and mTOR activity, reversing the principal dysregulations considered the most proximal events in ADPKD pathogenesis, making CaSR a possible candidate as therapeutic target.

## Introduction

Autosomal Dominant Polycystic Kidney Disease (ADPKD) is the fourth leading cause of end stage renal disease (ESRD) in adults, characterized by the progressive, bilateral growth and enlargement of fluid-filled cysts in kidneys that leads to a decline in renal function. It has a frequency of 1:400–1:1000^1^ and 50% of adult PKD patients will require dialysis or kidney transplantation by their 6th decade. ADPKD is a dominant inherited disease caused by loss-of-function mutations in the *PKD1* or *PKD2* gene, encoding polycystin-1 (PC1) or polycystin-2 (PC2), respectively^2^. PKD1 is responsible for 85% of the cases in clinically-affected individuals (ADPKD1) and is associated with a more severe clinical course, while mutations in *PKD2* are present in the remaining 15% of the patients (ADPKD2), who generally show a milder renal functional decline and a lower renal complication rate.

During the past few years, understanding of ADPKD pathogenesis has been considerably deepened, nevertheless the function of the polycystins and the molecular mechanisms underlying cysts development are still poorly understood. Polycystins belong to a family of eight proteins containing transmembrane domains that form a heteromeric molecular complex in the plasma membrane and cilia^3^. PC1 is localized to the primary cilium and to the cell junctions where it probably functions as a receptor and/or adhesion molecule. PC2 is a calcium-permeable nonselective cation channel, expressed on the primary cilium, endoplasmic reticulum, and the plasma membrane. PC1 and PC2 interact to form the PC complex, which localizes to the primary cilia and acts as a mechanosensor that controls calcium influx through the plasma membrane, induced by mechanical stimuli^4,5^. PC1 and PC2 are also known to regulate intracellular calcium release from the endoplasmic reticulum (ER) through their interaction with the inositol 1,4,5-trisphosphate receptor (IP_3_R)^6–8^. In conditionally immortalized, plasma membrane-permeabilized human proximal tubule epithelial cells, the simultaneous expression of both polycystins amplifies the IP_3_-induced calcium release, while PC1 alone or PC2 alone has no effect^9^. Despite the diversity of conclusions reached in the numerous studies analyzing the mechanisms involved in intracellular calcium regulation operated by PC1 and PC2, most of them are consistent with the hypothesis that polycystins by themselves and through their interaction with other calcium channels in the endoplasmic reticulum prevent the depletion of intracellular stores, maintaining the amplitude of physiological calcium oscillations^10,11^. The similar effect of both polycystins on the intracellular calcium homeostasis explains why loss-of-function mutations in the PKD1 or in the PKD2 genes both cause ADPKD.

Calcium signaling dysregulation is strictly correlated to another ADPKD hallmark represented by elevated cAMP levels. Numerous animal models of PKD show increased content of cAMP in the kidney^12–16^, an effect also observed in cholangiocytes^17^, in vascular smooth muscle cells^18^, and in choroid plexus^19^. Several hypotheses have connected the increased levels of cAMP in PKD tissues to the dysregulation of intracellular calcium signaling, specifically correlating the reduced cytosolic calcium to both cAMP synthesis and hydrolysis. The decrement in cytosolic calcium is supposed to cause the activation of the calcium-inhibitable adenylate cyclase 6 (AC6), to directly inhibit calcium/calmodulin dependent phosphodiesterase 1 (PDE1) and to increase the levels of cyclic guanosine monophosphate, thus inhibiting indirectly the cyclic guanosine monophosphate-inhibitable PDE3^14,20^. Increased cAMP levels are also attributable to the dysfunction occurred in the PC ciliary complex where the disruption of the PC2-mediated calcium entry, activates AC5/6 and inhibits phosphodiesterase 4C (PDE4C)^21^. Another study proposed the activation of the calcium-inhibitable AC6 due to the oligomerization and translocation of STIM1 to the plasma membrane, caused by the ER calcium stores depletion^22^. cAMP increase causes protein Kinase A (PKA) activation, which leads to ERK-mediated phosphorylation of tuberin (the TSC2 gene product), inducing upregulation of the mammalian target of rapamycin, mTOR, implicated in the pro-proliferative pathway^23–25^. This process has also been linked to the abnormal transcriptional activation of aerobic glycolysis and intracellular ATP accumulation, allowing liver kinase B1 inhibition which, together with AMP-activated protein kinase (AMPK) inhibition, may further enhance mTOR signaling^26,27^.

The two crucial dysregulations in ADPKD, intracellular calcium and cAMP levels, are two pathways both regulated in the kidney by the activation of the extracellular calcium-sensing receptor (CaSR)^28,29^. CaSR is a G-protein-coupled receptor, originally cloned from the bovine parathyroid gland and successively identified in various organs^30^. Besides the parathyroid gland, the key CaSR-expressing organs are intestine, bone, and kidney^31,32^. The CaSR senses changes in extracellular calcium concentrations and regulates parathyroid hormone (PTH) secretion and renal tubular calcium reabsorption to maintain serum calcium levels within the normal range. Increased serum calcium concentrations activate CaSR expressed in the parathyroid gland eliciting a G_q_-protein cascade that activates the phospholipase C (PLC) pathway and prevents exocytosis of PTH^33^. In the proximal tubule, CaSR is expressed on the apical membrane and its activation evoked by an increment in luminal calcium, decreases PTH-induced intracellular cAMP accumulation by inhibiting adenylate cyclase 6^29,34^.

In a recent work, we have shown that conditionally immortalized human proximal tubular epithelial cells (ciPTEC), isolated from urine of a healthy subject, express a functional CaSR sensitive to its physiological agonist, calcium, and its positive allosteric modulator, NPS-R568, resulting in a release of calcium from intracellular stores^35^. Moreover, receptor activation was found to cause a significant decrease in intracellular cAMP levels^35^.

In the present study, we investigated the expression of CaSR in ciPTEC with stable knockdown of PKD1 as well as in ciPTEC isolated from urine of a PKD1 patient. Of note, we show that CaSR activation increased cytosolic calcium, and reduced intracellular cAMP levels and mTOR activity thus improving the principal dysregulations of signaling molecules considered the most proximal events in the pathogenesis of ADPKD, making CaSR a possible therapeutic target candidate.

## Results

### Expression and functional characterization of CaSR in ciPTECwt and ciPTEC-PC1KD

**Figure 1:**
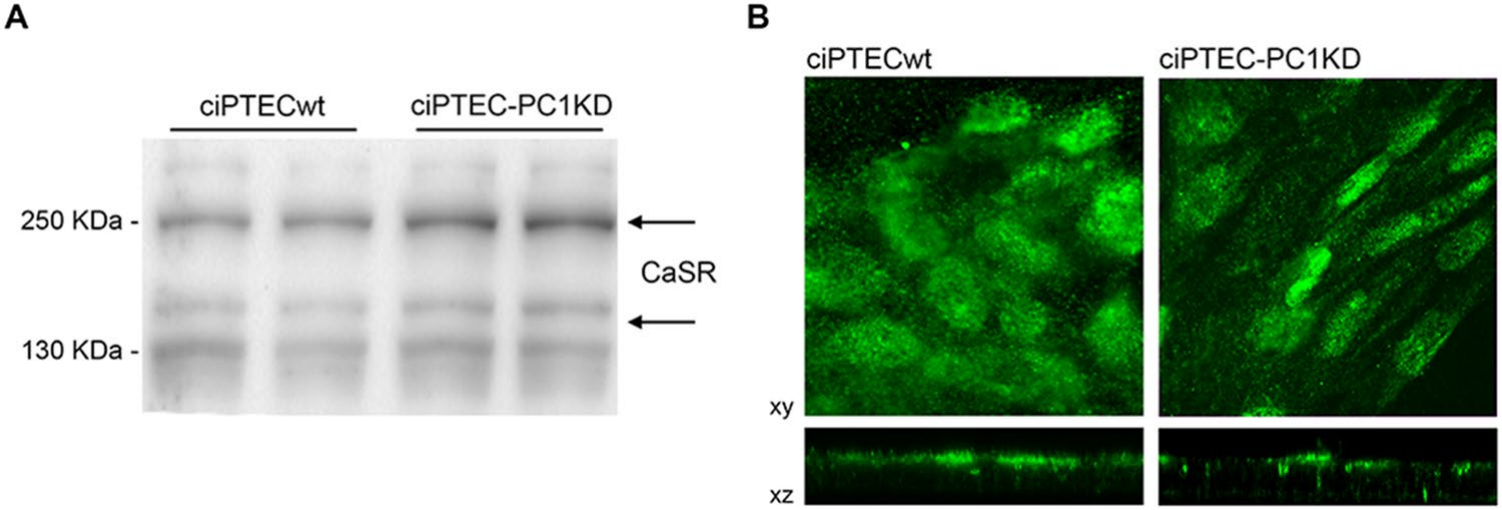
Endogenous CaSR expression and localization in ciPTECwt and ciPTEC-PC1KD. (**A**) Immunodetection of CaSR in homogenates of ciPTECwt and ciPTEC-PC1KD, after 11 days of maturation at 37 °C. Specific anti-CaSR antibodies revealed both CaSR forms at 130 and 250 kDa, corresponding to the monomeric and mature receptor. The figure shows a representative blot. (**B**) Immunofluorescence localization of CaSR in polarized ciPTEC, showing its predominant apical plasma membrane localization.

The endogenous CaSR expression in ciPTEC stably knocked down for polycystin-1^9^ (ciPTEC-PC1KD) and in the wild type clone (ciPTECwt) was evaluated by Western blotting. Both cell type expressed the monomeric form at 130 kDa, the glycosylated monomeric one at 160 kDa, and the dimeric receptor at 250 kDa (Fig. 1A). Moreover, no difference in CaSR expression levels was observed in the two cell lines (data not shown). Immunofluorescence CaSR localization in monolayers of polarized ciPTEC revealed an apical plasma membrane expression of the receptor in both cell lines as it occurs in native renal proximal tubule epithelial cells (Fig. 1B).

**Figure 2:**
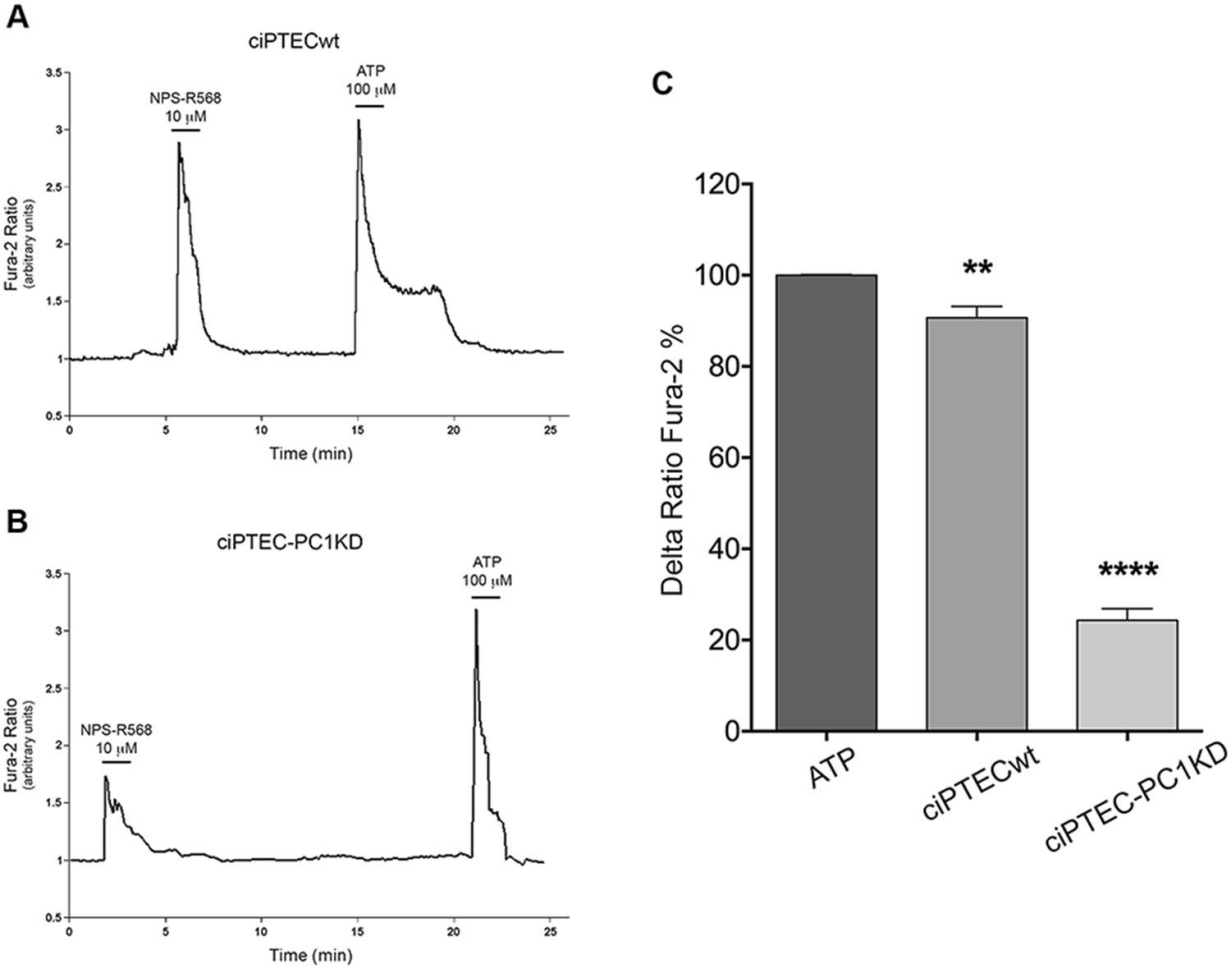
Effects of CaSR positive allosteric modulator, NPS-R568, on (Ca^2+^)_i_ levels. ciPTECwt (**A**) and ciPTEC-PC1KD (**B**) were grown for 11 days at 37 °C and stimulated with NPS-R568 10 μM and ATP 100 μM. Fluorescence ratio 340/380 nm was recorded. Each trace is representative of 4 different experiments with similar results. (**C**) Fluorescence ratio 340/380 nm was recorded and responses to NPS-R568 were calculated as the percentage of changes in fluorescence (Delta Ratio Fura-2%), normalized to the fluorescence ratio observed in the presence of the ATP stimulus (100%). Histogram shows a significant lower intracellular calcium increase in ciPTEC-PC1KD compared to ciPTECwt. Data were analyzed with One-way ANOVA followed by Newman-Keuls multiple comparisons test and are expressed as means ± SEM (**P < 0.01 vs. ATP; ****P < 0.0001 vs. ATP or ciPTECwt).

In a previous study, we have provided the first evidence that ciPTEC isolated from urine of a healthy volunteer endogenously express a functional CaSR^35^. Here, we show that both ciPTECwt and ciPTEC-PC1KD also express a functional receptor. Cells were loaded with 6 μM Fura-2 AM and stimulated with the positive allosteric CaSR modulator NPS-R568 (10 μM) known to increase the sensitivity of the receptor for calcium^36^. CaSR stimulation caused a significant increase in Ca^2+^_i_ in both ciPTECwt and ciPTEC-PC1KD as showed in Fig. 2A and B, reporting a representative time course of fluorescence responses. Nevertheless, Ca^2+^_i_ increase in ciPTECwt was significantly higher with respect to the one observed in ciPTEC-PC1KD. Statistical analysis of the fluorescence responses revealed that cytosolic calcium levels were 90.68 ± 2.47% (vs. ATP 100%, n = 40) in ciPTECwt and 24.32 ± 2.59% (vs. ATP 100%, n = 47) in ciPTEC-PC1KD, P<0.0001 (Fig. 2C).

### Intracellular and ER calcium content in ciPTECwt and ciPTEC-PC1KD

**Figure 3:**
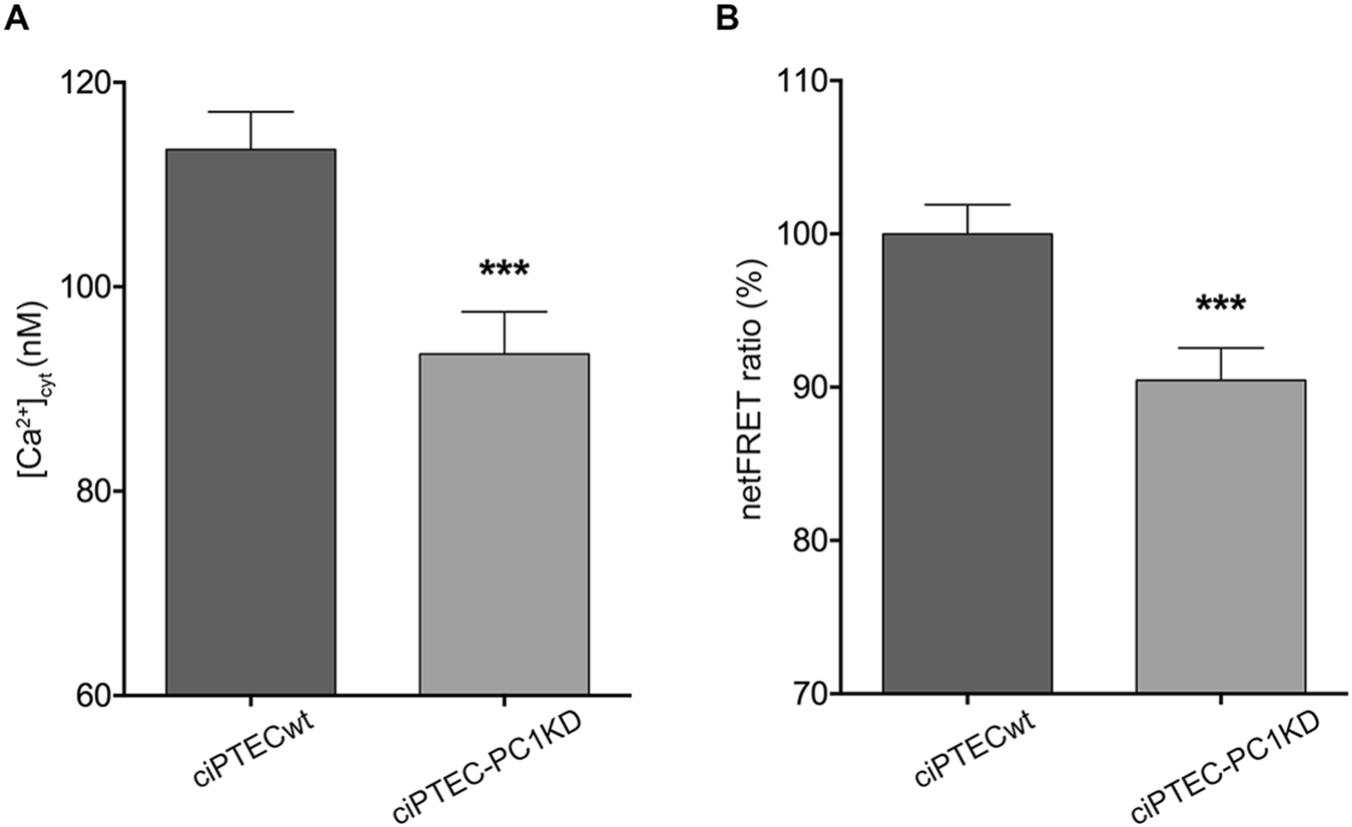
Calcium content in ciPTECwt and ciPTEC-PC1KD. (**A**) Intracellular calcium concentrations measurement obtained by calcium calibration experiments. Cells were loaded with 6 μM Fura-2 AM for 15 min at 37 °C. Free cytosolic Ca^2+^ was calculated accordingly to Grynkiewicz formula. Histogram shows a significant lower intracellular calcium concentration in ciPTEC-PC1KD compared to ciPTECwt. Data were analyzed by unpaired t-test and are expressed as means ± SEM (***P = 0.0004). (**B**) Evaluation of Ca^2+^_ER_ with ER-targeted Cameleon (D1ER) FRET probe. Binding of calcium to calmodulin sequence results in an intramolecular rearrangement of the probe leading to an increase in netFRET signal. Histogram (means ± SEM, ***P = 0.0008) compares changes in normalized FRET (netFRET) ratio between ciPTECwt and ciPTEC-PC1KD, analyzed by unpaired t-test.

Polycystin dysfunctions have been proven to cause a reduction in steady state calcium levels^37^ which contributes to cyst formation^38,39^. It has been shown that cultured epithelial cells derived from human ADPKD cysts have a basal intracellular calcium content approximately 20 nM lower than normal human kidney (NHK) cells^38^. Therefore, we first measured the cytosolic calcium levels at rest in our cell models. Calcium calibration experiments revealed a significant lower [Ca^2+^]_i_ in ciPTEC-PC1KD compared to ciPTECwt (ciPTEC-PC1KD = 93.44 ± 4.13 nM, n = 107; ciPTECwt = 113.4 ± 3.7 nM, n = 95; P = 0.0004), confirming calcium dysregulation in ADPKD cells and in animal models (Fig. 3A).

Since it is known that PC complex disruption has implications in abnormal ER calcium depletion^11,40^, FRET experiments were performed to measure calcium levels in the ER. ciPTEC-PC1KD had significant lower ER calcium levels compared with ciPTECwt (Fig. 3B), consistent with the lower increase in intracellular calcium after CaSR stimulation showed in Fig. 2C (ciPTEC-PC1KD = 90.46 ± 2.09%, n = 140, vs. ciPTECwt = 100%, n = 152; P = 0.0008).

### CaSR activation decreases cAMP levels in ciPTEC-PC1KD

**Figure 4:**
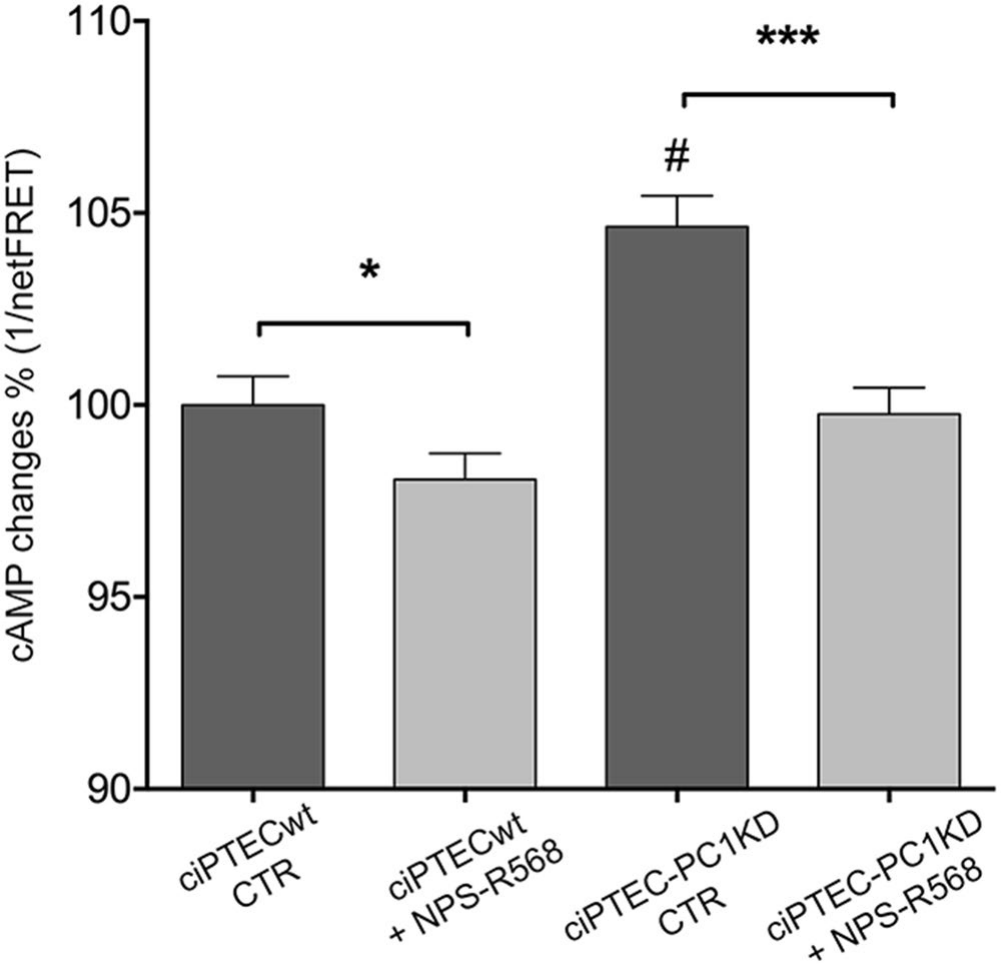
Evaluation of cAMP levels in ciPTECwt and ciPTEC-PC1KD by FRET analysis. At resting, ciPTEC-PC1KD presented a significant higher cAMP content with respect to ciPTECwt. Interestingly, CaSR stimulation elicited by NPS-R568 10 μM induced a significant decrease in cAMP content in ciPTEC-PC1KD, restoring levels comparable to ciPTECwt at basal conditions. All data were analyzed by One-way ANOVA followed by Newman-Keuls multiple comparisons test and are expressed as means ± SEM (*P < 0.01 ciPTECwt CTR vs. ciPTECwt + NPS-R568; ***P < 0.0001 ciPTEC-PC1KD CTR vs. ciPTEC-PC1KD + NPS-R568; ^#^P < 0.0001 ciPTEC-PC1KD CTR vs. ciPTECwt CTR).

As in native cells from cysts^41^, significantly higher cAMP levels were found under basal conditions in ciPTEC-PC1KD with respect to ciPTECwt (ciPTEC-PC1KD = 104.6 ± 0.81%, n = 96, vs. ciPTECwt = 100%, n = 114; P<0.0001) (Fig. 4). Interestingly, treatment of cells with the CaSR positive allosteric modulator NPS-R568 (10 μM for 30 min) induced a significant decrease in ciPTEC-PC1KD intracellular cAMP content (ciPTEC-PC1KD + NPS-R568 = 99.76 ± 0.68%, n = 115), approaching the levels observed in ciPTECwt at basal conditions. This result represents the first evidence that cells knocked down for PC1 expressing an endogenous functional CaSR, respond to the calcimimetic NPS-R568 with a significant reduction in cAMP intracellular levels, thus attenuating one of the pivotal dysregulations characterizing ADPKD.

### mTOR dysregulation is improved by CaSR activation in ciPTEC-PC1KD

**Figure 5:**
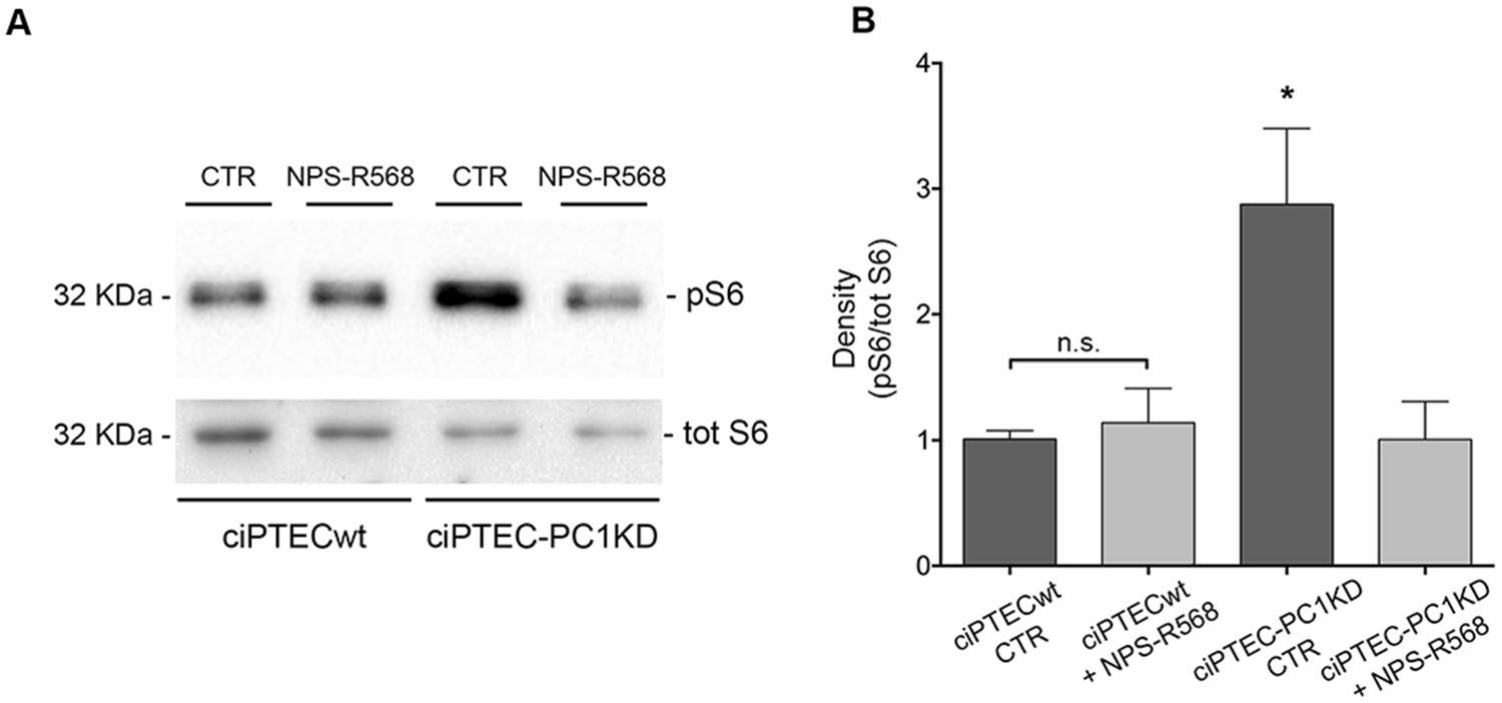
NPS-R568 effect on mTOR expression and activity in ciPTECwt and ciPTEC-PC1KD, analyzed by pS235/236-S6 (pS6) evaluation. (**A**) Equal amount of proteins from cells (30 μg) were immunoblotted for total S6 and pS6. The figure shows representative blots. (**B**) Signals were semiquantified by densitometry. Statistical analysis performed with One-way ANOVA followed by Newman-Keuls multiple comparisons test (means ± SEM, *P < 0.001 ciPTEC-PC1KD CTR vs. ciPTECwt CTR or ciPTECwt + NPS-R568 or ciPTEC-PC1KD + NPS-R568) revealed that pS6 expression, higher in ciPTEC-PC1KD compared to ciPTECwt, was restored at the same levels of ciPTECwt at rest by the treatment with NPS-R568.

mTOR activity was investigated by the evaluation of the phosphorylated forms of its downstream effector, the S6 ribosomal protein, and its upstream effector, AMP-activated protein kinase (AMPK). Specifically, S6 is used as a marker of mTORC1 pathway activation^42^. mTOR signaling activates p70S6 kinase which in turn phosphorylates Ser235, Ser236, Ser240 and Ser244 of S6^43^. Anti-pS6 (Ser235/236) antibodies were used for pS6 immunodetection (Fig. 5). pS6 expression at rest was significantly higher in ciPTEC-PC1KD compared with ciPTECwt (ciPTECwt = 1 ± 0.06, n = 10; ciPTEC-PC1KD = 2.88 ± 0.6, n = 8; P<0.001). Importantly, CaSR activation by NPS-R568 (10 μM for 30 min), reduced S6 protein levels in ciPTEC-PC1KD to those observed in ciPTECwt at basal conditions (ciPTEC-PC1KD + NPS-R568 = 1.07 ± 0.3, n = 10). CaSR stimulation did not affect pS6 levels in ciPTECwt with respect to untreated cells.

**Figure 6:**
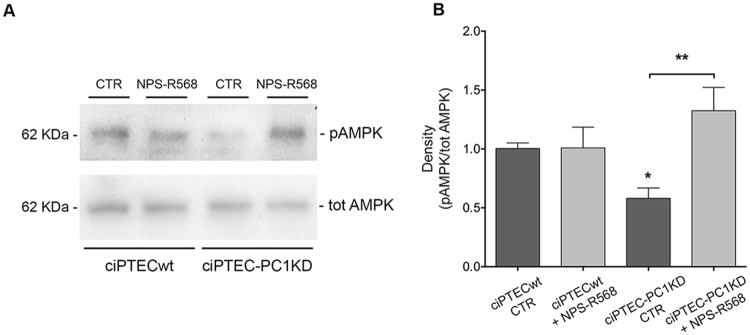
AMPK phosphorylation levels under NPS-R568 effect in ciPTECwt and ciPTEC-PC1KD. (**A**) Equal amount of proteins (30 μg) were immunoblotted with antibodies specific for total AMPK or for pT172-AMPK (pAMPK). The figure shows representative blots. (**B**) Densitometric analysis and statistical studies performed with One-way ANOVA followed by Newman-Keuls multiple comparisons test (means ± SEM) revealed that pAMPK levels in ciPTEC-PC1KD were significantly lower with respect to ciPTECwt. CaSR stimulation with NPS-R568 restored levels comparable to ciPTECwt at rest (*P < 0.0001 ciPTEC-PC1KD CTR vs. ciPTECwt CTR; **P < 0.001 ciPTEC-PC1KD + NPS-R568 vs. ciPTEC-PC1KD CTR).

Conversely, mTOR is known to be inhibited by AMPK^44^, and AMPK activity is decreased in PC1 knocked down cells compared with control. We evaluated AMPK in ciPTEC by Western blotting experiments (Fig. 6) examining its phosphorylation at Thr172 in the α subunit (pAMPK). The phosphorylated levels of AMPK were significantly lower in ciPTEC-PC1KD compared to ciPTECwt (ciPTECwt = 1 ± 0.05, n = 15; ciPTEC-PC1KD = 0.58 ± 0.09, n = 15; P<0.0001). After treatment with NPS-R568 (10 μM for 30 min), pAMPK levels in ciPTEC-PC1KD were reversed to levels comparable to wt cells (ciPTEC-PC1KD + NPS-R568 = 1.32 ± 0.2, n = 15). No changes were observed in pAMPK expression in ciPTECwt after NPS-R568 treatment with respect to untreated cells.

**Figure 7:**
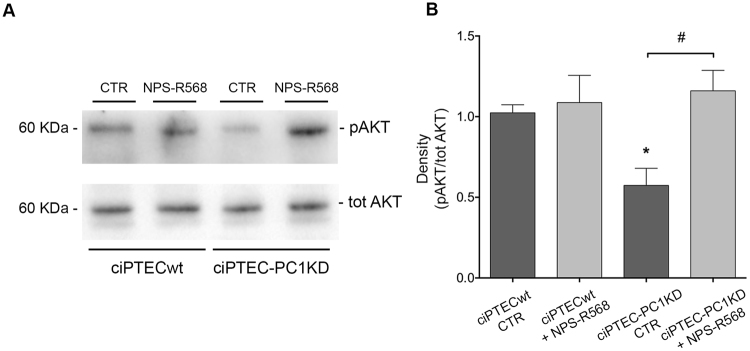
NPS-R568 effect on Akt expression in ciPTECwt and ciPTEC-PC1KD. (**A**) Equal amount of proteins from cells (30 μg) were immunoblotted for total Akt and Akt phosphorylated at Ser473 (pAkt). The figure shows representative blots. (**B**) Densitometric and statistical analysis (means ± SEM, *P < 0.01 ciPTEC-PC1KD CTR vs. ciPTECwt CTR; ^#^P < 0.01 ciPTEC-PC1KD + NPS-R568 vs. ciPTEC-PC1KD CTR) demonstrated significantly lower pAkt levels in ciPTEC-PC1KD compared to ciPTECwt. CaSR stimulation with NPS-R568 increased pAkt to levels detected in ciPTECwt at rest. One-way ANOVA followed by Newman-Keuls multiple comparisons test was used to analyze data.

**Figure 8:**
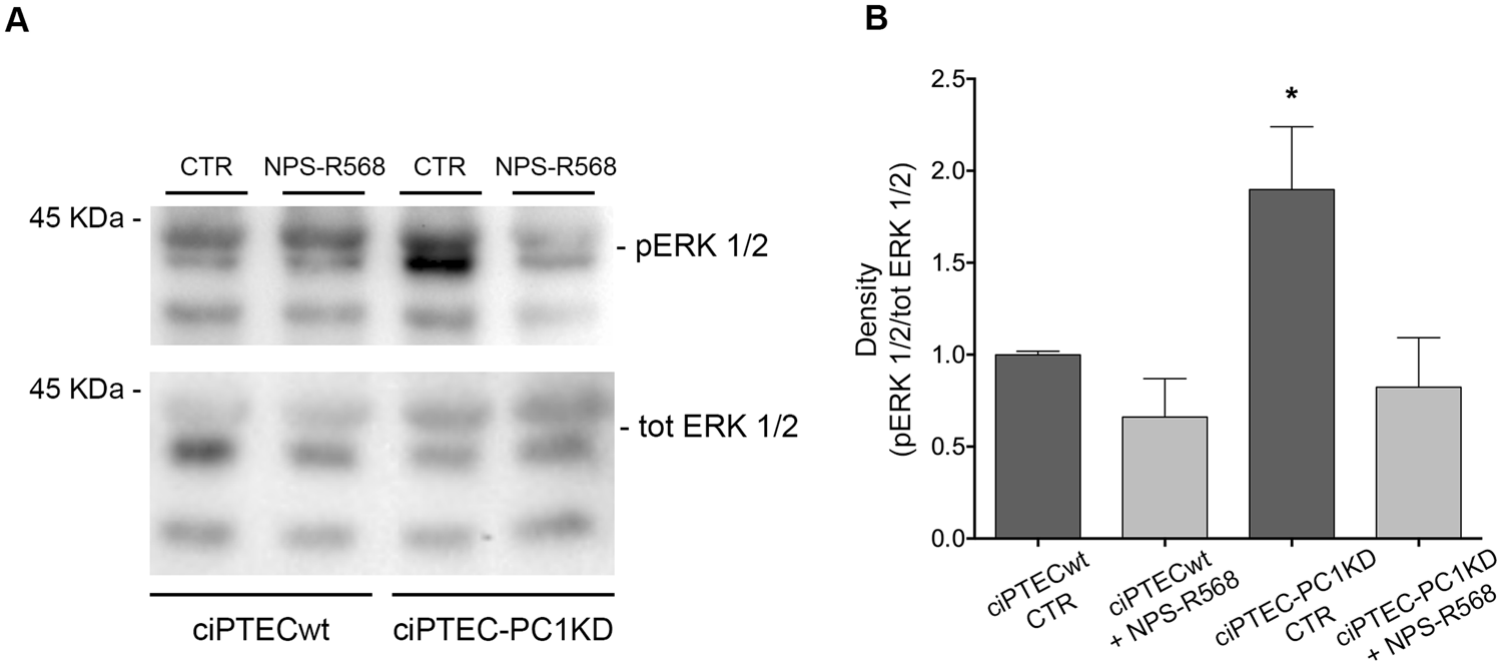
Effect of CaSR stimulation on ERK 1/2 expression in ciPTECwt and ciPTEC-PC1KD. (**A**) Equal amount of proteins from cells (30 μg) were immunoblotted for total ERK 1/2 and ERK 1/2 phosphorylated at Thr185/Tyr187 (pERK 1/2). The figure shows representative blots. (**B**) Signals were semiquantified by densitometry. Statistical analysis (means ± SEM, *P < 0.05 ciPTEC-PC1KD CTR vs. ciPTECwt CTR or ciPTEC-PC1KD + NPS-R568 vs. ciPTEC-PC1KD CTR) revealed significantly higher pERK 1/2 expression in ciPTEC-PC1KD compared to ciPTECwt. CaSR stimulation with NPS-R568 restored the same levels of pERK 1/2 to levels detected in ciPTECwt at rest. One-way ANOVA followed by Newman-Keuls multiple comparisons test was used to analyze data.

AMPK regulates mTOR also through the direct phosphorylation of the tumor suppressor TSC2^44^, resulting in mTOR inhibition. We therefore evaluated the serine/threonine kinase Akt, an upstream negative regulator of TSC2, whose activation mediates cell growth, proliferation, and survival. In ciPTEC-PC1KD cells, the levels of phosphorylated Akt (pAkt, Ser-473) were significantly reduced with respect to wt (ciPTECwt CTR = 1 ± 0.05, ciPTEC-PC1KD CTR = 0.57 ± 0.11, n = 8; P<0.01) (Fig. 7). Interestingly, CaSR stimulation with NPS-R568, by increasing cytosolic calcium concentration, elicited an increase in pAkt in ciPTEC-PC1KD with respect to ciPTECwt, restoring the basal content (ciPTEC-PC1KD + NPS-R568 = 1.16 ± 0.13, n = 8). Since increased Akt activity inhibits cAMP-dependent B-Raf/ERK, ERK/pERK were next evaluated. pERK 1/2 levels were found increased in ciPTEC-PC1KD compared to ciPTECwt (ciPTECwt = 1 ± 0.02, ciPTEC-PC1KD = 1.89 ± 0.34, n = 7; P < 0.05) (Fig. 8). Interestingly, CaSR activation decreased pERK 1/2 expression in ciPTEC-PC1KD to the levels observed in wt cells (ciPTEC-PC1KD + NPS-R568 = 0.82 ± 0.3, n = 7).

### CaSR activation reverses PKD1 dysregulations in ciPTEC isolated from an ADPKD1 patient

**Figure 9:**
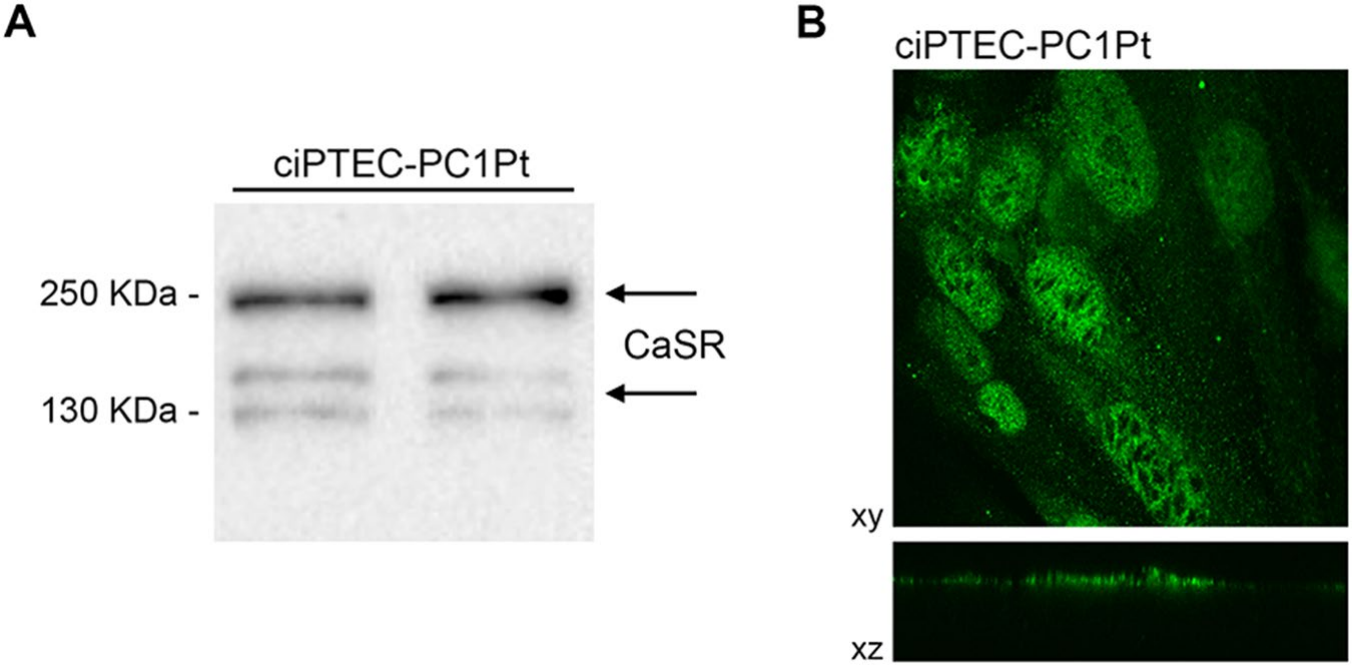
Endogenous CaSR expression and localization in ciPTEC-PC1Pt. (**A**) After 11 days of maturation at 37 °C, cells were homogenized and CaSR immunodetection was performed. Specific anti-CaSR antibodies revealed the expression of both CaSR forms at 130 and 250 kDa, corresponding to the monomeric and mature receptor. The figure shows a representative blot. (**B**) Immunofluorescence staining of CaSR, showing its apical plasma membrane expression in a confluent monolayer of ciPTEC-PC1Pt.

The effect of CaSR activation in ciPTEC-PC1Pt was next evaluated. The absence of immunoreactive PC1 band in the ADPKD patient (10.032) was previously reported^45^. CaSR expression was confirmed by Western blotting experiments, showing the presence of both the forms of the receptor, the monomeric and mature protein (Fig. 9A), and by confocal analysis in monolayers of polarized ciPTEC-PC1Pt proving the apical localization of CaSR (Fig. 9B).

**Figure 10:**
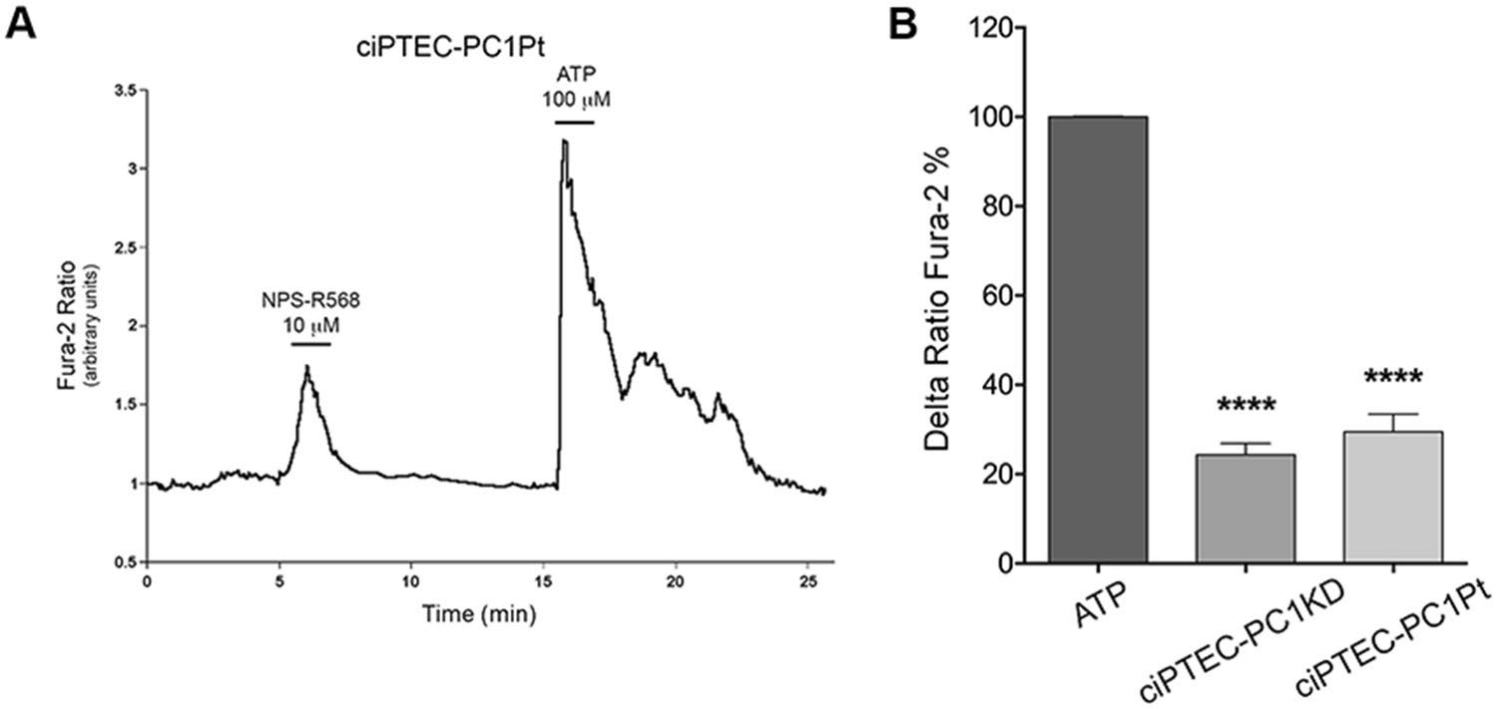
Functional characterization of endogenous CaSR in ciPTEC-PC1Pt. (**A**) Cells were grown for 11 days at 37 °C and stimulated with NPS-R568 10 μM and ATP 100 μM. Fluorescence ratio 340/380 nm was recorded. Each trace is representative of 3–4 different experiments with similar results. (**B**) Histogram shows that NPS-R568 stimulation in ciPTEC-PC1Pt elicited an intracellular calcium increase comparable to the one obtained in ciPTEC-PC1KD. Fluorescence ratio 340/380 nm was recorded and responses to NPS-R568 were calculated as the percentage of changes in fluorescence (Delta Ratio Fura-2%), normalized to the fluorescence ratio observed in the presence of the ATP stimulus (100%). Data were analyzed with One-way ANOVA followed by Newman-Keuls multiple comparisons test and are expressed as means ± SEM (****P < 0.0001 vs. ATP).

The functional expression of the receptor present in ciPTEC-PC1Pt was next evaluated by single-cell epifluorescence imaging. Cells were loaded with 6 μM Fura-2 AM and treated with 10 μM NPS-R568 (Fig. 10A). As ciPTEC-PC1KD, ciPTEC-PC1Pt responded to CaSR activation with a significant increase in cytosolic calcium (29.51 ± 3.93% vs. ATP 100%, n = 38, P < 0.0001) (Fig. 10B). Interestingly, the calcium levels observed were comparable with those reported in ciPTEC-PC1KD, suggesting a similar dysregulation in calcium homeostasis in cells isolated from ADPKD1 patient urine as well.

**Figure 11:**
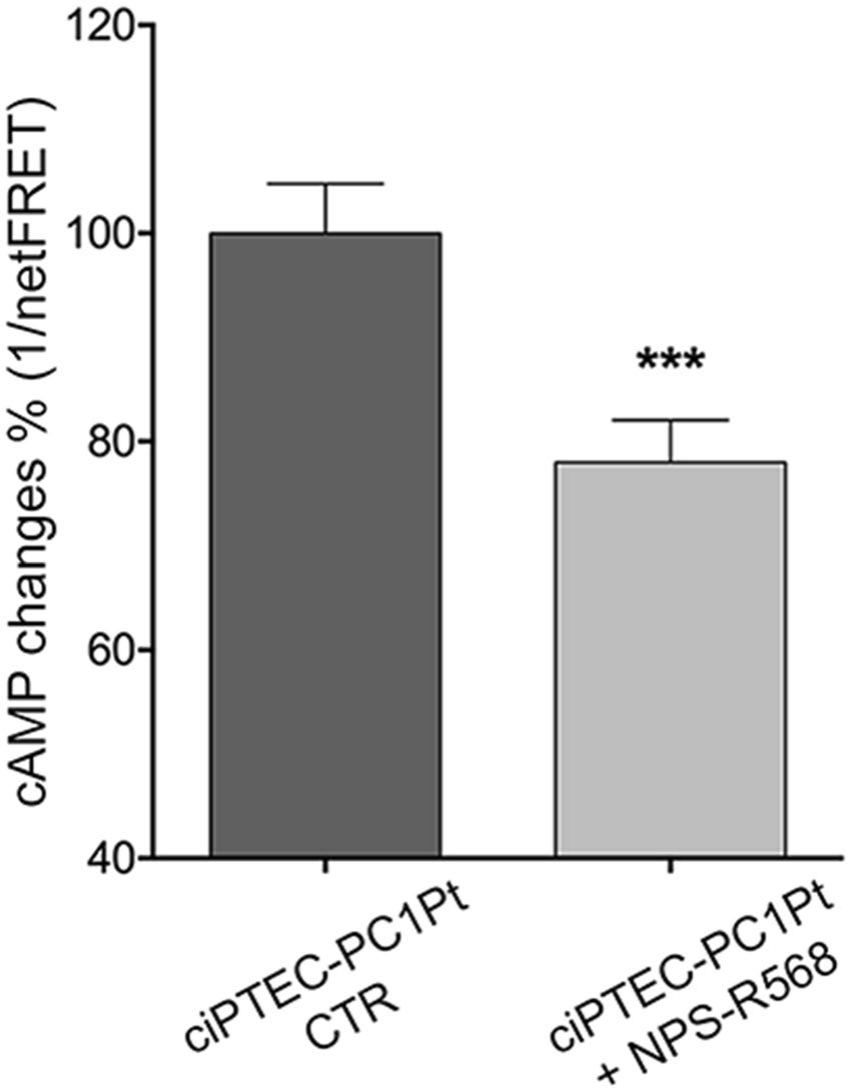
Effect of CaSR activation on cAMP content in ciPTEC-PC1Pt. FRET experiments revealed that CaSR stimulation with NPS-R568 10 μM significantly reduced cAMP levels in ciPTEC-PC1Pt with respect to untreated cells. Data are expressed as means ± SEM (***P = 0.0006).

Of note, as observed in ciPTEC-PC1KD, CaSR stimulation with NPS-R568 caused a significant reduction of cAMP levels also in ciPTEC-PC1Pt (ciPTEC-PC1Pt + NPS-R568 = 78.01 ± 4.04%, n = 120, vs. ciPTEC-PC1Pt CTR = 100%, n = 140; P = 0.0006; Fig. 11).

**Figure 12:**
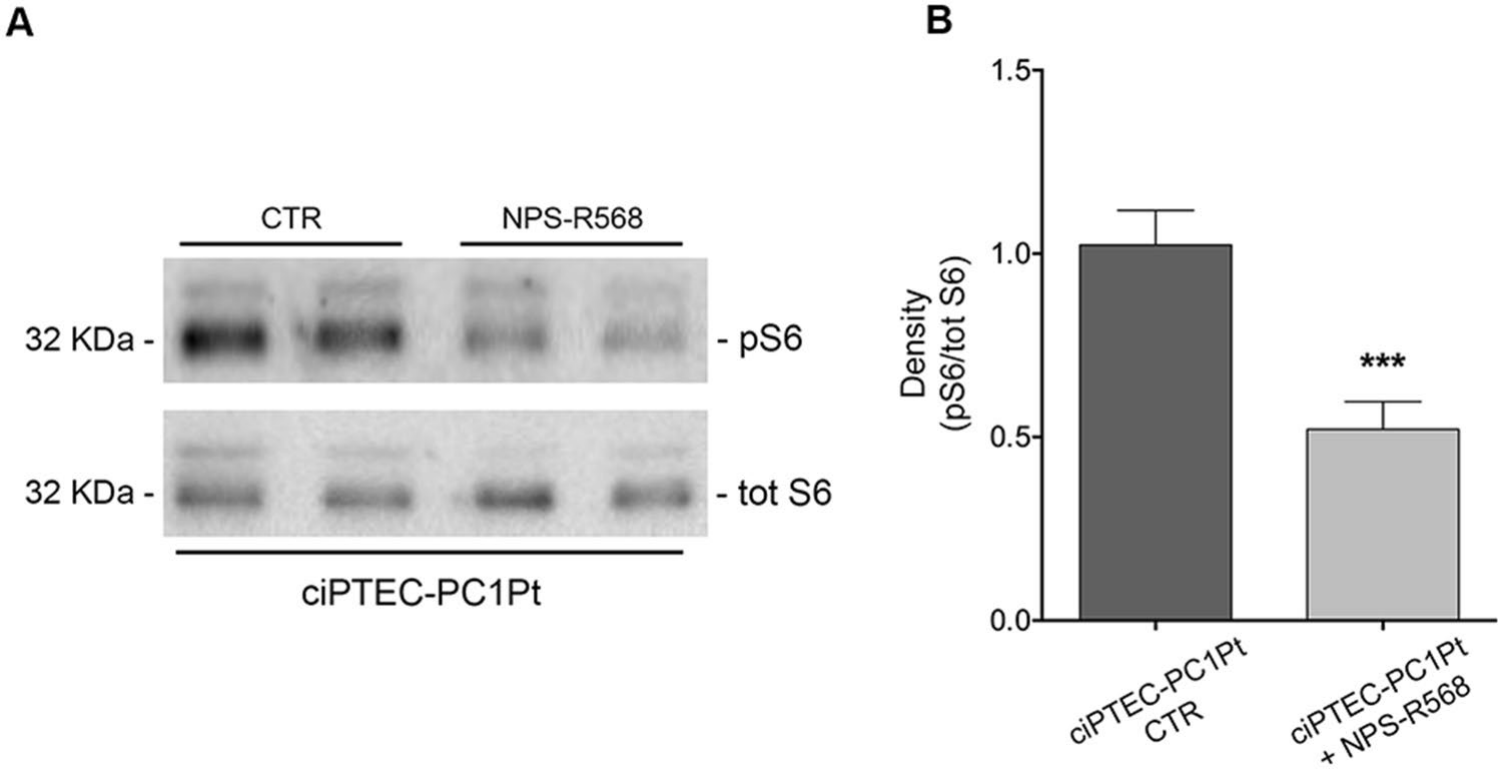
Effect of CaSR stimulation on mTOR expression and activity in ciPTEC-PC1Pt, evaluated by pS235/236-S6 (pS6) levels analysis. (**A**) Equal amount of proteins from cells (30 μg) were immunoblotted for total S6 and pS6. The figure shows representative blots. (**B**) Densitometric and statistical analysis performed with unpaired t-test (means ± SEM, ***P = 0.0004) revealed that pS6 expression was significantly decreased by the treatment with NPS-R568 compared to untreated cells.

**Figure 13:**
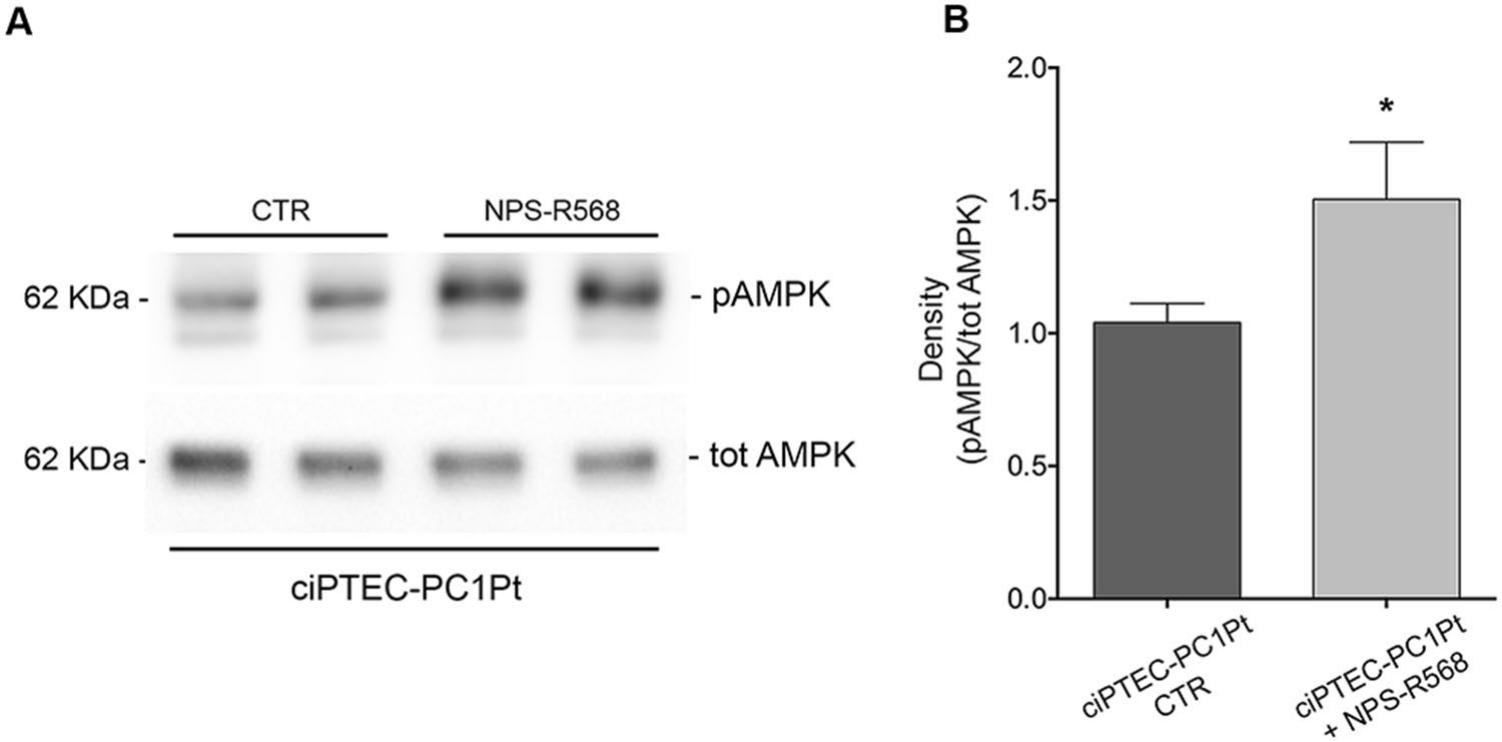
NPS-R568 stimulation effect on AMPK phosphorylation levels in ciPTEC-PC1Pt. (**A**) Equal amount of proteins (30 μg) were immunoblotted with antibodies specific for total AMPK or for pT172-AMPK (pAMPK). The figure shows representative blots. (**B**) Signals were semiquantified by densitometry. Statistical analysis (means ± SEM, *P = 0.02) demonstrated that CaSR activation elicited a significant increase in pAMPK levels with respect to untreated cells. Data were analyzed by unpaired t-test.

The expression and activity of mTOR and AMPK were also evaluated in ciPTEC-PC1Pt. CaSR activation with NPS-R568 caused a significant decrease in pS6 levels compared to unstimulated cells which reflects a lower mTOR activity in response to CaSR activation in this cell line (ciPTEC-PC1Pt + NPS-R568 = 0.52 ± 0.08, n = 10, vs. ciPTEC-PC1Pt CTR = 1 ± 0.09, n = 10; P = 0.0004; Fig. 12). Conversely, CaSR stimulation resulted in an increased AMPK activity as demonstrated by the significantly higher levels of the phosphorylated form in NPS-R568 treated cells (ciPTEC-PC1Pt + NPS-R568 = 1.51 ± 0.22, n = 8, vs. ciPTEC-PC1Pt CTR = 1 ± 0.07, n = 11; P = 0.02; Fig. 13).

## Discussion

The major results obtained in this work can be summarized as follows: *a*. ciPTEC with stable knockdown of PKD1 or isolated from the urine of an ADPKD1 patient endogenously express functional CaSR; *b*. both cell lines showed the same dysregulations in intracellular calcium, cAMP and mTOR pathways reported in animal models of ADPKD1^12,14,20,46–50^ and human polycystic kidneys^38,51^; c. these dysregulations were reversed by activation of CaSR with the allosteric modulator NPS-R568. These results indicate that CaSR may represent a therapeutic target in Autosomal Dominant Polycystic Kidney Disease 1.

The first evidence that ciPTEC isolated from urine of a healthy volunteer endogenously express a functional CaSR was recently provided by our group^35^. We showed that CaSR activation causes a decrease in cytosolic cAMP content and an increase in intracellular calcium attributable to CaSR coupling to G_q_, resulting in PLC activation and IP_3_-dependent release of calcium from intracellular stores^35^. In the aim of exploring, at cellular level, the potentiality of CaSR as therapeutic target in the treatment of ADPKD, we considered, as valuable model system, ciPTEC obtained by immortalizing cells exfoliated from urine sediments of an ADPKD1 patient and ciPTEC obtained from healthy subject, knocked down for PKD1.

The complexity of the renal cyst formation still makes ADPKD a poorly understood disease, nevertheless it is known to involve cell clonal proliferation, increased apoptosis, abnormal epithelial cell phenotype, extracellular matrix alterations and inflammation^52,53^. Recent evidence suggests that for cystogenesis to occur, there is no requirement of a complete loss of PC1 or PC2 function, but their functionality must be reduced to a certain threshold level, which marks the correspondence between PC1 dosage and rate of disease severity^4^. In the renal collecting duct, mutations in PKD1 or PKD2 are associated with a decrease in intracellular calcium and increase in cAMP with consequent activation of PKA exposing collecting duct principal cells to the constant tonic effect of vasopressin, activating downstream signaling pathways responsible for impaired tubulogenesis, cell proliferation, increased fluid secretion, and interstitial inflammation^54^.

The pivotal role of cAMP in the pathogenesis of ADPKD represents a key point for treatment strategies rationale to lower its levels in cystic tissues. Clinical trials of vasopressin receptor 2 (V2R) antagonists have shown encouraging results. In fact, several studies demonstrated that V2 receptor antagonists (mozavaptan and/or tolvaptan) attenuate the progression of PKD in *cpk* mice and *pcy* mouse models of nephronophthisis (NPHP), ARPKD (PCK rats), and PKD2 (*Pkd2*^WS25/−^ mice)^14,47,48,55^. The V2R antagonist tolvaptan, by inhibiting the binding of vasopressin to the receptor, promotes aquaresis and lowers cAMP synthesis, slowing progression of renal cysts also in humans as shown in randomized placebo-controlled clinical trials^56–59^.

However, since the V2R is not present in the proximal tubule, tolvaptan would have no effect on cysts originating from this segment and a therapy directed to the proximal tubular cysts would probably improve the suboptimal efficiency of tolvaptan therapy. Interestingly, a recent study^60^ showed that treatment of *pcy* mice (a NPHP orthologous animal model), with the calcimimetic NPS-R568, a positive allosteric CaSR modulator, inhibited progression of cysts growth and renal fibrosis. NPS-R568 was less effective in later-stage NPHP but did significantly reduce kidney weight^60^. In an interesting work, Gattone and coworkers^61^ showed that NPS-R568, administrated to male Cy/+ rats from 20 to 38 weeks of age, an age when CKD is well established, reduced the development of renal cysts and ameliorated the kidney cystic disease. In contrast, another study evaluating the effect of CaSR activation with NPS-R568 in animal models orthologous to human ADPKD and/or ARPKD (Autosomal Recessive Polycystic Kidney Disease), reported no detectable effect on cystogenesis but possible beneficial effect on interstitial fibrosis^15,16,62,63^. A possible explanation for the lack of calcimimetic effect is that the administration of NPS-R568 resulted in hypocalcemia and therefore it is likely that the effects of NPS-R568 on intracellular calcium and cAMP are negated by the reduction in extracellular calcium.

In the present study, we show the cellular effect of CaSR activation with NPS-R568 on the most proximal events in ADPKD pathogenesis, calcium and cAMP, in ciPTEC of human proximal tubule origin stably knocked down for PKD1 or isolated from the urine of an ADPKD1 patient. Both the cell lines expressed the monomeric and the mature glycosylated forms of CaSR that localized at the apical plasma membrane as in native proximal tubule cells. At basal conditions, ciPTEC-PC1KD showed lower cytosolic calcium concentrations with respect to ciPTECwt, reporting one of the two major dysregulations observed in ADPKD1 animal models. Of note, functional experiments showed that CaSR activation, elicited by NPS-R568 stimulation, caused an increase in cytosolic calcium both in ciPTEC-PC1KD and ciPTEC-PC1Pt both having calcium values significantly lower than ciPTECwt. In a previous work, we already showed that CaSR expressed in ciPTEC couples to G_q_ and its activation results in PLC activation and IP_3_-dependent release in intracellular calcium, likely from endoplasmic reticulum^35^. Several studies highlighted the primary role of the PC complex interaction with other calcium channels expressed in the ER in preventing the depletion of intracellular stores, especially of the ER itself^10,11,40^. Here, we demonstrated a lower calcium content, at steady state, in the ER of ciPTEC-PC1KD compared to ciPTECwt, sustaining the hypothesis that the lower intracellular calcium increase observed in ciPTEC-PC1KD and ciPTEC-PC1Pt was due to a lower calcium content in the ER, attributable to the presence of a loss-of-function mutation in PC1, which leads to PC complex disruption and dysfunction. Moreover, as mentioned before, intracellular calcium decrement in ADPKD has been correlated to cAMP increase also observed in ciPTEC-PC1KD. Interestingly, NPS-R568 treatment elicited a significant decrease in cAMP content in ciPTEC-PC1KD and in ciPTEC-PC1Pt, restoring levels comparable to ciPTECwt at basal conditions. We show here that CaSR activation had a positive effect also on mTOR activity, which is upregulated in cystic kidney epithelial cells showing altered cell proliferation. In normal kidney, PC1 and TSC1/TSC2 interaction inhibits mTOR, preventing an abnormal activation of the proliferative pathway. By contrast in ADPKD, PC1 dysfunction precludes the TSC1/TSC2 complex formation promoting mTOR activation, through a mechanism involving the cAMP-dependent B-Raf/ERK pathway^64,65^. Specifically, in human primary ADPKD cyst epithelial cells it has been shown that increase in intracellular cAMP activates PKA leading to B-Raf and hence MEK and ERK activation^25^. In addition, it has been reported that disruption of intracellular calcium mobilization operated by calcium channel blockers was associated with decreased activity of Akt, a negative regulator of B-Raf, resulting in activation of the B-Raf/ERK pathway^66^.

In line with these results, lower levels of pAkt and increased pERK have been found in ciPTEC-PC1KD. Of note, NPS-R568 treatment induced an increase in pAkt and a reduction in pERK levels. It has to be pointed out that pAkt is an upstream positive regulator of mTOR through TSC2 inhibition expected to increase mTOR signaling. However, the overall reduction in mTOR signaling observed in ciPTEC-PC1KD cells in response to NPS-R568 can be due to the observed activation of AMPK known to reduce mTOR signaling through TSC2 phosphorylation^44,67^ which might overcome the pAkt effect.

AMPK is activated by Ca^2+^/calmodulin-dependent protein kinase-β (CaMKKβ) in response to an increase in cytosolic calcium concentration^68^. We report here that AMPK is downregulated in ciPTEC-PC1KD and NPS-R568 stimulation caused a significant increase in pAMPK either in ciPTEC-PC1KD and ciPTEC-PC1Pt.

Therefore, CaSR showed a regulatory effect on multiple defective pathways either in ciPTEC-PC1KD and ciPTEC-PC1Pt, potentiating the relevance that CaSR stimulation might have in improving ADPKD dysregulations.

To summarize, we have shown that selective CaSR activation in human ciPTEC carrying PKD1 mutation increases intracellular calcium, reduces cAMP and mTOR activity thus reversing the principal dysregulations of the molecules considered the most proximal events in the pathogenesis of ADPKD. Although ciPTEC are of proximal origin, it has to be underlined that CaSR is expressed in all nephron segments with an apical localization in the proximal tubule and in the collecting duct, and a basolateral distribution in the thick ascending limb where it displays the highest expression^34^. Therefore, *in vivo*, calcimimetics are expected to act in the entire nephron. Moreover, being CaSR also express in liver^69^, calcimimetics may also act on liver cysts also found in ADPKD patients. Investigating *in vitro* effects of the treatment with calcimimetics in human renal cells deriving from ADPKD patient has potentially important implications at several levels. Firstly, it can contribute to elucidate in much greater detail the pathophysiology of ADPKD. Secondly, it can have a direct impact on a novel way for ADPKD treatment as CaSR is expressed in all nephron segments where it counteracts the hormones acting through cAMP/PKA pathways making CaSR a promising candidate for therapeutic intervention in ADPKD.

## Methods

### Materials

All chemicals were purchased from Sigma (Sigma-Aldrich, Milan, Italy). Fura-2 AM was obtained from Molecular Probes (Life Technologies, Monza, Italy). NPS-R568 was kindly gifted by Amgen (Amgen Dompé S.p.a., Milan, Italy). Media for cell culture were from Lonza (Lonza s.r.l., Milan, Italy).

### Antibodies

Monoclonal CaSR antibody recognizing amino-acid 15–29 at the extracellular N-terminus was from Sigma-Aldrich, Milan, Italy. Rabbit anti-Phospho-S6RP (Ser235/236), anti-Tot S6RP, anti-Phospho-AMPK (Thr172 of its α subunit) and anti-Tot AMPK antibodies were purchased from Cell Signaling Technology (Beverly, Massachusetts, USA). Mouse anti-ERK 1/2 and rabbit anti-Phospho-ERK 1/2 (Thr185/Tyr187) were from Merck Millipore (Darmstadt, Germany). Secondary goat anti-rabbit, goat anti-mouse and goat anti-mouse IgG biotin antibodies were purchased from Sigma-Aldrich, Milan, Italy. Streptavidin-488 conjugate was from Alexa Fluor (Molecular Probes, Eugene, Oregon, USA).

### Generation of ciPTEC knocked down for polycystin-1 or from ADPKD1 patient

ciPTEC were generated as described by Wilmer and colleagues^70^. Primary cells were cultured by collecting mid-stream urine within 5 h after collection. Urine sediment was resuspended in DMEM Ham’s F12 medium supplemented with 10% fetal bovine serum (FBS), 100 IU/ml penicillin, 100 mg/ml streptomycin, ITS (5 μg/ml insulin, 5 μg/ml transferrin and 5 ng/ml selenium), 36 ng/ml hydrocortisone, 10 ng/ml epidermal growth factor (EGF) and 40 pg/ml triiodothyronine. The suspension was placed at 37 °C in a 5% CO_2_ incubator.

Primary cells were immortalized as previously described^70^. Briefly, cells were infected with SV40T and hTERT vectors, containing respectively geneticin (G418) and hygromycin resistance^71,72^. Subconfluent cell layers were transferred to 33 °C and selected by using G418 (400 μg/ml) and hygromycin B (25 μg/ml) for 10 days. Stable knocked down ciPTEC for polycystin-1 (ciPTEC-PC1KD) were obtained transducing a cloned ciPTEC line (ciPTECwt) of a healthy individual (34.8) by adding lentiviral vectors encoding miR-shRNA directed against polycystin-1, cloned in tandem (pCHMWS Bsd 2xmiRNA PKD1), to the culture medium^9^. Transduced cells were selected using 10 g/ml blasticidin. Alternatively, ciPTEC derived from an ADPKD1 patient (10.032) with known germline PKD1 mutation (ciPTEC-PC1Pt) were isolated as described^45,70^. Experiments were performed prior cellular maturation for 11 days at 37 °C. The reduced expression of PC1 was showed by Mekahli and coworkers which biochemically characterized these cell lines^9,45^.

### Immunofluorescence Microscopy

Immunofluorescence localization of CaSR in polarized ciPTEC was performed as previously described^35,73^. Cells were incubated with antibodies diluted in block solution containing 2% (w/v) bovine serum albumin (BSA) and 0.1% (v/v) tween-20 in HBSS against the calcium-sensing receptor (CaSR, 1:800 dilution) at 4 °C overnight. Following treatment with secondary rabbit-anti-mouse-biotin antibodies followed by Streptavidin-488, samples were mounted on glass slides with Mowiol. Images were obtained with a confocal microscope Leica TCS SP2 (Leica Microsystems, Heerbrugg, Switzerland).

### Cell Preparations

ciPTEC were seeded onto 100-mm dishes and grown at 37 °C for 11 days, then were lysed in Cell Fractionation Buffer (20 mM NaCl, 130 mM KCl, 1 mM MgCl_2_, 10 mM Hepes, pH 7.5) in the presence of proteases (1 mM PMSF, 2 mg/ml leupeptin and 2 mg/ml pepstatin A) and phosphatases (10 mM NaF and 1 mM sodium orthovanadate) inhibitors. Cellular debris was removed by centrifugation at 12,000 × g for 20 min at 4 °C. The supernatants were collected and used for immunoblotting studies.

### Gel Electrophoresis and Immunoblotting

ciPTEC lysates were separated on 10% bis-tris acrylamide gels under reducing conditions. Protein bands were electrophoretically transferred onto Immobilon-P membranes (Millipore Corporate Headquarters, Billerica, USA) for Western blot analysis, blocked in TBS-Tween-20 containing 5% BSA and incubated with primary antibodies O/N. Anti-CaSR was used at 1:800 dilution, anti-Phospho-S6RP and anti-Tot S6RP were used at 1:1000 dilution, anti-Phospho-AMPK and anti-Tot AMPK at 1:500 dilution. Immunoreactive bands were detected with secondary antibodies conjugated to horseradish peroxidase (HRP) obtained from SantaCruz Biotechnologies (Tebu-Bio, Milan, Italy). Membranes were developed using Super Signal West Pico Chemiluminescent Substrate (Pierce, Rockford, USA) with Chemidoc System (Bio-Rad Laboratories, Milan, Italy). Representative figures are shown. Densitometry analysis was performed with Scion Image. Data were summarized in histograms with GraphPad Prism (Graphpad Software Inc. La Jolla, CA, USA).

### Video-Imaging Experiments

ciPTEC were grown on 25-mm glass coverslips at 37 °C for 11 days, then were loaded with 6 μM Fura-2 AM for 15 min at 37 °C in DMEM. Ringer’s Solution was used to perfuse cells during the experiment containing 120 mM NaCl, 4 mM KCl, 15 mM NaHCO_3_, 1 mM MgCl_2_, 15 mM Hepes, 0.5 mM NaH_2_PO_4_, 10 mM Glucose, 1 mM CaCl_2_, 0.5 mM Na_2_HPO_4_, 0.4 mM MgSO_4_, pH 7.4 (modified by^9,36^). Measurements were performed using an inverted microscope (Nikon Eclipse TE2000-S microscope) equipped for single cell fluorescence measurements and imaging analysis. The sample was illuminated through a 40 × oil immersion objective (NA = 1.30). The Fura-2 AM loaded sample was excited at 340 and 380 nm. Emitted fluorescence was passed through a dichroic mirror, filtered at 510 nm (Omega Optical, Brattleboro, VT, USA) and captured by a cooled CCD camera (Cool SNAP HQ, Photometrics). The ratio of fluorescence intensities at 340 and 380 nm was plotted using Metafluor software (Molecular Devices, MDS Analytical Technologies, Toronto, Canada).

For the experiments at steady state, intracellular calcium level was calibrated and then calculated as described by Grynkiewicz^74^. Briefly, intracellular calcium concentration (Ca^2+^)_i_ was determined from the emission fluorescence ratio of the two excitation wavelengths accordingly to the formula (Ca^2+^)_i_ = Kd · Q(R − R_min_)/(R_max_ − R), where Kd (224 nM) indicates the dissociation constant of Fura-2 AM for (Ca^2+^)_i_ and Q indicates the ratio of the fluorescence intensities (F) at the minimum and the maximum calcium concentration at 380 nm. Each sample was calibrated by the addition of 5 μM ionomycin in the presence of 1 mM EGTA (R_min_) followed by 5 μM ionomycin in 5 mM CaCl_2_ (R_max_).

### Fluorescence Resonance Energy Transfer (FRET) Measurements

To evaluate intracellular cAMP levels and endoplasmic reticulum (ER) calcium content, fluorescence resonance energy transfer (FRET) experiments were performed as described^35^. Briefly, ciPTEC were seeded onto 20-mm glass coverslips at 37 °C for 11 days. For cytosolic cAMP evaluation, cells were transiently transfected with a plasmid encoding the H96 probe containing cAMP binding sequence of Epac1 between CFP and cp173Venus-Venus^75^. For ER calcium levels measurements, cells were transiently transfected with a plasmid encoding the D1ER Cameleon^76^. Experiments were performed 48 hours post-transfection. Cells were left under basal condition or, for intracellular cAMP evaluation, also stimulated with NPS-R568 (10 μM for 30 min at 37 °C) in Ringer’s solution described above, containing 2 mM CaCl_2_.

FRET measurements were carried out using MetaMorph software (Molecular Devices, MDS Analytical Technologies, Toronto, Canada). CFP and YFP were excited at 436 and 500 nm, respectively; fluorescence emitted was measured at 480/40 nm for CFP and 535/30 nm for YFP and FRET. Corrected normalized FRET values were determined as already described^77,78^. Each image was corrected for CFP cross-talk and YFP cross-excitation. Therefore, netFRET = [IFRETbg − ICFPbg K_1_ − IYFPbg · (K_2_ − αK_1_)]/(1 − δK_1_) where IFRETbg, ICFPbg, and IYFPbg are the background-corrected pixel gray values measured in the FRET, CFP and YFP windows, respectively; K_1_, K_2,_ α and δ are calculated to evaluate the crosstalk between donor and acceptor. The integrated fluorescence density values of the images from each cell were analyzed using MetaMorph and Microsoft Excel software.

### Statistical analysis

One-way ANOVA followed by Newman-Keuls multiple comparisons test or t-test were used for the statistical analysis. All values are expressed as means ± SEM. A difference of P < 0.05 was considered statistically significant.

### Data availability statement

All data generated or analysed during this study are included in this published article.

### Ethical approval and informed consent

The current study is in accordance with the institutional ethical guidelines for obtaining human cell lines for research and was approved by the corresponding ethical committee at University Hospitals Leuven. An informed consent was obtained from all participants and/or their legal guardian/s.

## Electronic supplementary material

Supplementary Information
