# Supplementary material for: Activation of Calcium-Sensing Receptor increases intracellular calcium and decreases cAMP and mTOR in PKD1 deficient cells
Source: Sci Rep. 2018 Apr 9;8:5704. doi: 10.1038/s41598-018-23732-5 (PMC5890283; doi:10.1038/s41598-018-23732-5)
Supplement: Supplementary file 1 — Supplementary Information [file 41598_2018_23732_MOESM1_ESM.pdf]

Activation of Calcium-Sensing Receptor increases intracellular calcium and decreases cAMP and mTOR in PKD1 deficient cells.

Annarita Di Mise[1,*], Grazia Tamma[1,5], Marianna Ranieri[1], Mariangela Centrone[1], Lambertus van den Heuvel[2], Djalila Mekahli[3,4], Elena N. Levtchenko[3,4], Giovanna Valenti[1,5,6,*]

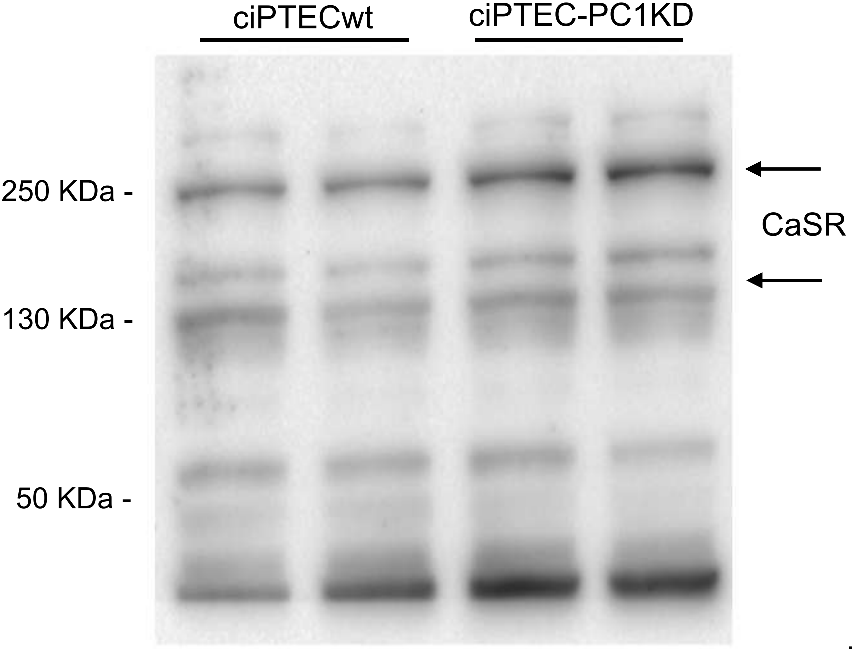

Fig. 1A

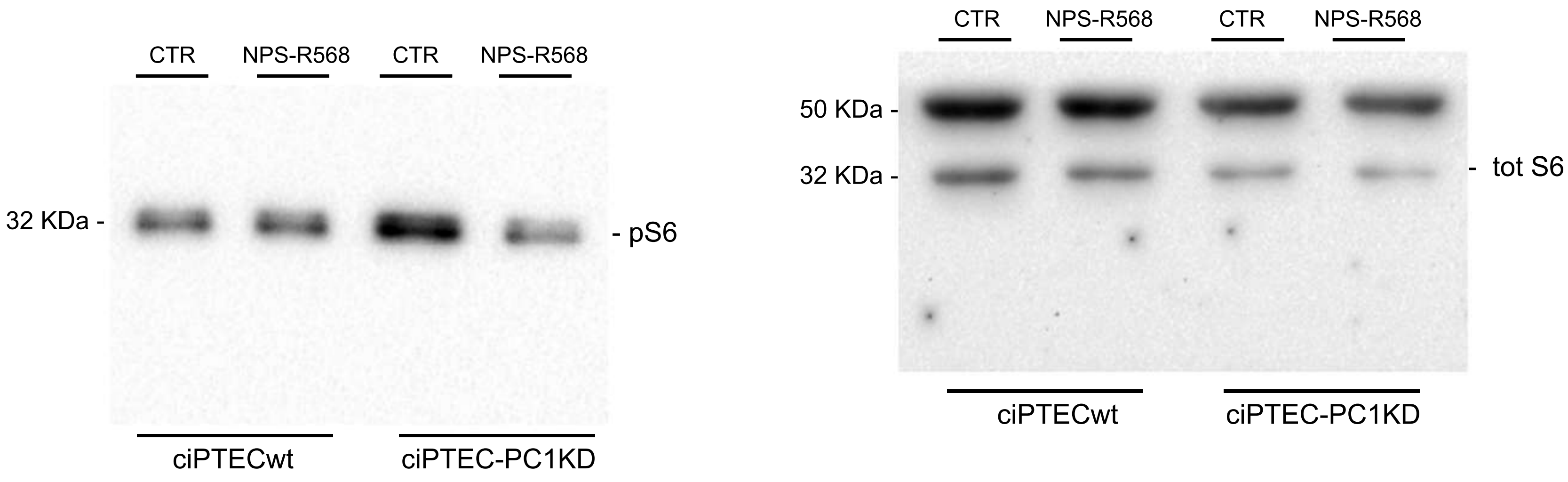
CTR
NPS-R568
CTR
NPS-R568
32 KDa -
- pS6
ciPTECwt
ciPTEC-PC1KD
CTR
NPS-R568
CTR
NPS-R568
50 KDa -
32 KDa -
- tot S6
ciPTECwt
ciPTEC-PC1KD

Fig. 5A

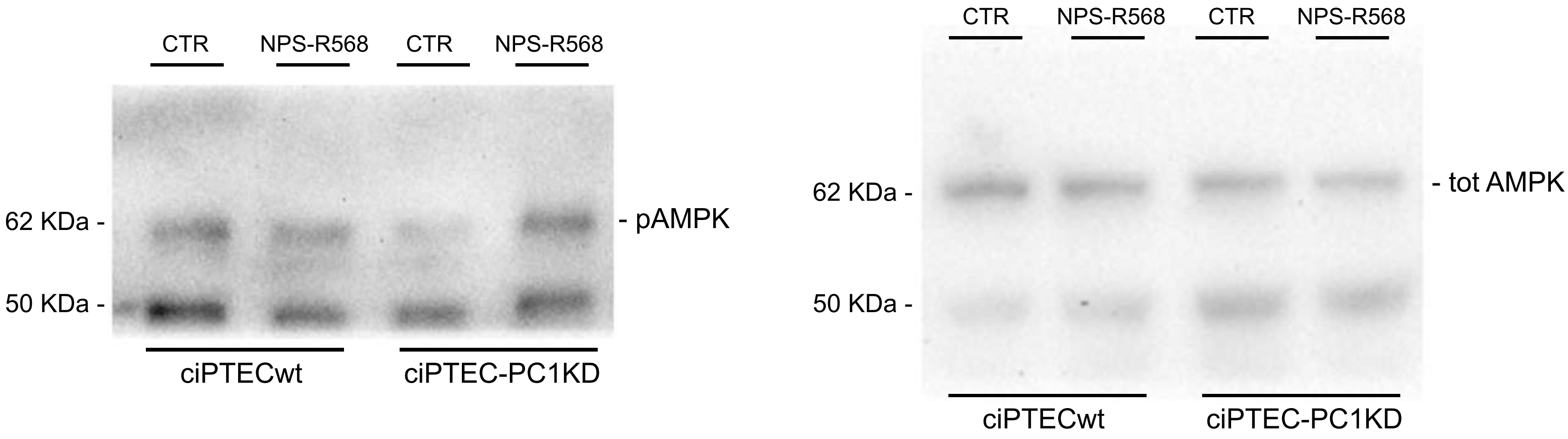
CTR
NPS-R568
CTR
NPS-R568
62 KDa -
50 KDa -
- pAMPK
ciPTECwt
ciPTEC-PC1KD
CTR
NPS-R568
CTR
NPS-R568
62 KDa -
50 KDa -
- tot AMPK
ciPTECwt
ciPTEC-PC1KD

Fig. 6A

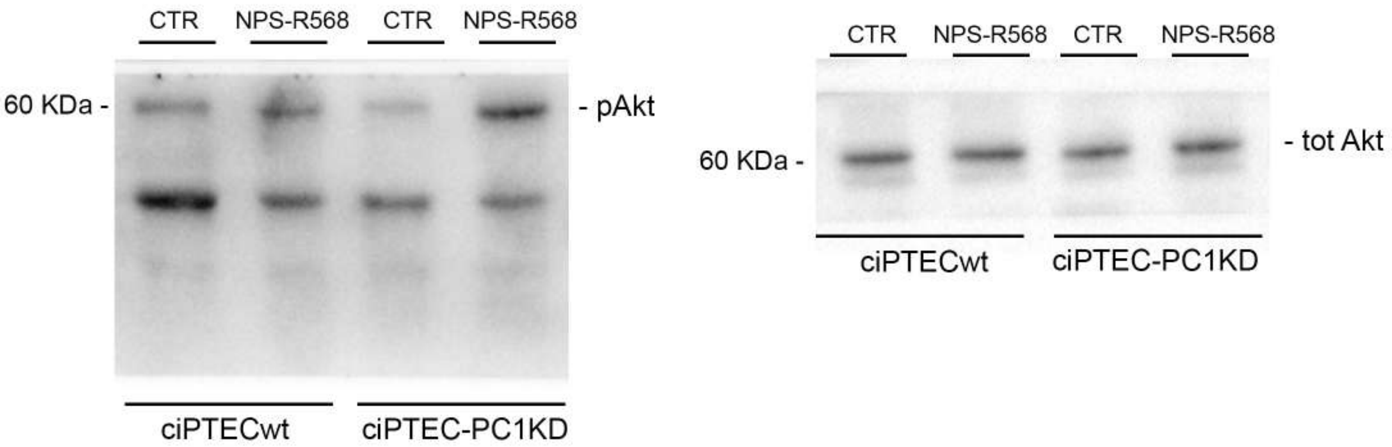
CTR
NPS-R568
CTR
NPS-R568
60 KDa -
- pAkt
ciPTECwt
ciPTEC-PC1KD
CTR
NPS-R568
CTR
NPS-R568
60 KDa -
- tot Akt
ciPTECwt
ciPTEC-PC1KD

Fig. 7A

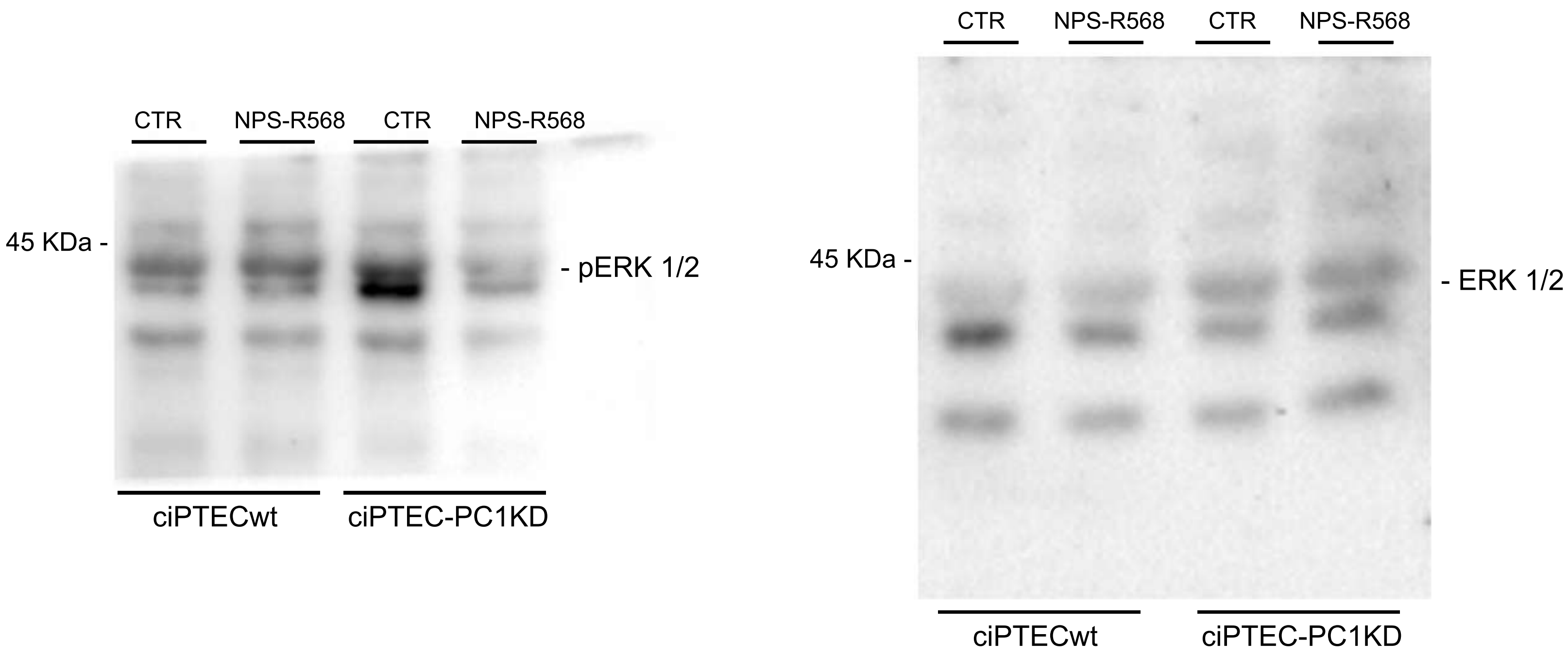
CTR
NPS-R568
CTR
NPS-R568
45 KDa -
- pERK 1/2
ciPTECwt
ciPTEC-PC1KD
CTR
NPS-R568
CTR
NPS-R568
45 KDa -
- ERK 1/2
ciPTECwt
ciPTEC-PC1KD

Fig. 8A

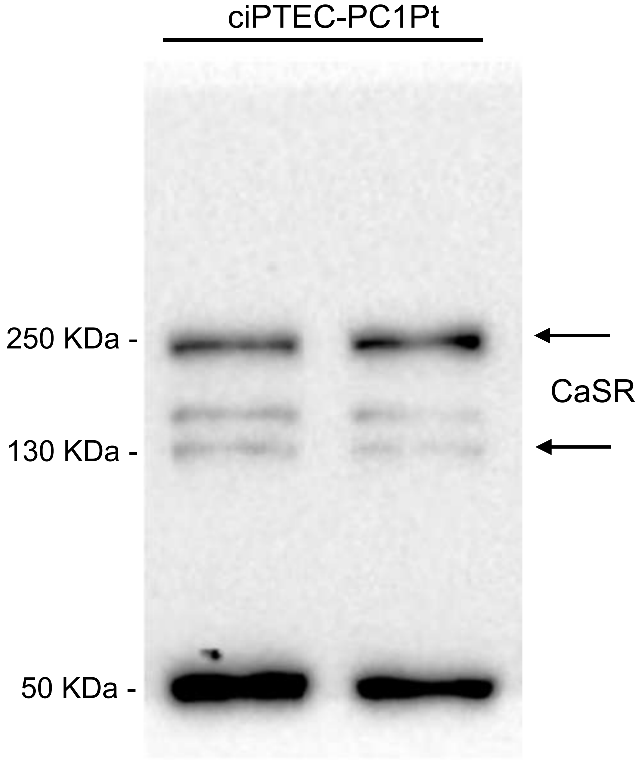

Fig. 9A

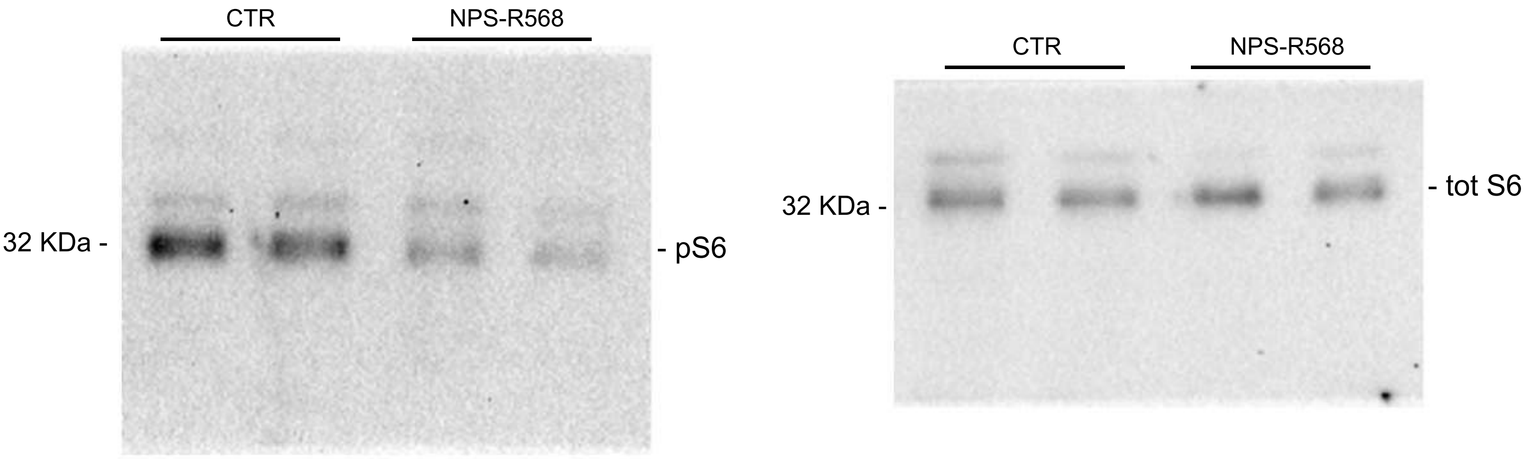

Fig. 12A

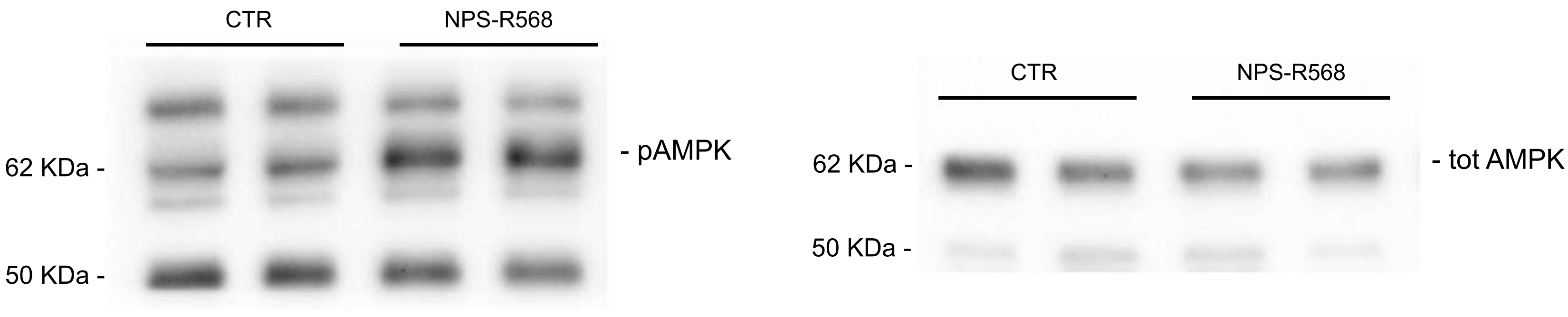
CTR
NPS-R568
62 KDa -
50 KDa -
- pAMPK
CTR
NPS-R568
62 KDa -
50 KDa -
- tot AMPK

Fig. 13A
